# Current News

**Published:** 1901-03

**Authors:** 


### Current News.

### TRI-STATE DENTAL MEETING—A LEGAL NOTICE.

KNOW all men by these presents that on the fourth, fifth, and sixth days of the sixth month, in the year one thousand nine hundred and some, Annie Dominoes, in the City of Indianapolis, State of Felicity, otherwise known as Indiana, with the Banks of the Wabash not far away, and several other banks a good deal closer, there will be held, convened, and, as you might say, congregated, a galaxy, or assemblage of practising dentists, for the purpose of meeting, getting together, and associating themselves in conclave, to hear papers and discussions, see clinics and exhibits, and otherwise inform, enlighten, refresh, and amuse themselves, in a just and lawful manner, according to that clause in the Constitution of the United States which insures every citizen of this republic protection in the pursuit of happiness.

And know all you same men, by these further Christmas presents, that this is the third, tertiary, or ternary, joint or amalgamated meeting of the State Societies of Ohio, Michigan, and Indiana, which said joint meetings take place or occur every third year, beginning at the commencement, and may be called triennial in their being as well as triangulate. And, therefore, as the first of these meetings, held at Detroit in 1895, was, is, and will be known as the O! I'm meeting, and the second, held at Put-in-Bay, in 1898, was raised and called the Mi, O! meeting, be it hereby and hereon ordained, specified, ordered, and otherwise understood that this coming Tri-State Dental Meeting of the State Societies of Ohio, Michigan, and Indiana, to be held at Indianapolis, Ind., June 4, 5, and 6, 1901, shall be known officially as the O, Mi! meeting.

You are all invited to come and break bread with us.

If there is any further information desired that is not imparted in the above, it may be accumulated by communicating with

GEO. E. HUNT,
*Chairman.*

INDIANAPOLIS, IND.

### DENTAL SURGEONS IN THE ARMY.

THE following is part of Section 18 of the new Army Law, approved February 2, 1901, and relating especially to the employment of dentists in the army as "contract dental surgeons:"

"That the Surgeon-General of the Army, with the approval of the Secretary of War, be, and he is hereby, authorized to employ and appoint dental surgeons to serve the officers and enlisted men of the Regular and Volunteer Army in the proportion of one dental surgeon to every one thousand of said Army, and not exceeding thirty in all. Said dental surgeons shall be employed as contract dental surgeons, under the terms and conditions applicable to army contract surgeons, and shall be graduates of standard medical or dental colleges, trained in the several branches of dentistry, of good moral and professional character, and shall pass a satisfactory professional examination: *Provided,* That three of the number of dental surgeons to be employed shall be first appointed by the Surgeon-General, with the approval of the Secretary of War, with reference to their fitness for assignment, under the direction of the Surgeon-General, to the special service of conducting the examinations and supervising the operations of the others, and for such special service an extra compensation of sixty dollars a month shall be allowed: *Provided further,* That dental college graduates now employed in the Hospital Corps, who have been detailed for a period of not less than twelve months to render dental service to the Army and who are shown by the reports of their superior officers to have rendered such service satisfactorily, may be appointed contract dental surgeons without examination."

The examiners appointed are J. S. Marshall, M.D., Chicago, Ill.; R. T. Oliver, D.D.S., Indianapolis, Ind.; and R. W. Morgan, D.D.S., Lynchburg, Va.

---

### NEW YORK STATE DENTAL SOCIETY.

THE annual meeting of the New York State Dental Society will be held on Wednesday and Thursday, May 8 and 9, in the assembly hall at Hotel Ten Eyck, Albany, N. Y.

The following essayists will present papers upon subjects to be announced: G. V. I. Brown, M.D., D.D.S., Wisconsin; E. S.

Talbot, M.D., D.D.S., Chicago, Ill.; W. E. Griswold, M.D., D.D.S., Denver, Col.; W. A. Purrington, LL.D., New York; H. D. Hatch, D.D.S., New York; A. R. Cooke, D.D.S., Syracuse, N. Y.

Members of the profession are cordially invited to be present. Head-quarters, Hotel Ten Eyck. Special rates, $3.50 per day.

Dr. W. A. White,
*Secretary.*

---

### HARVARD ODONTOLOGICAL SOCIETY.

At the annual election of the Harvard Odontological Society, at Boston, January 31, 1901, the following officers were elected for the ensuing year:

President, Joseph Totten Paul, D.M.D.; Recording Secretary, Robert Tucker Moffatt, D.M.D.; Corresponding Secretary, Arthur Henry Stoddard, D.M.D.; Treasurer, Lyman F. Bigelow, D.M.D.; Editor, Harry W. Haley, D.M.D.

*Executive Committee.*—Dr. Robert T. Moffatt, Chairman; Dr. William P. Cooke, Dr. Frank T. Taylor.

Robert T. Moffatt,
*Corresponding Secretary.*

---

### PENNSYLVANIA BOARD OF DENTAL EXAMINERS.

The Board of Dental Examiners of Pennsylvania will conduct examinations simultaneously in Pittsburg and Philadelphia, May 7 to 10.

Apply to Hon. James W. Latta, Secretary Dental Council, for examination papers and further information.

G. W. Klump,
*Secretary.*

Williamsport, Pa.

---

### SOUTHERN DENTAL SOCIETY OF NEW JERSEY.

At the annual meeting of the Southern Dental Society of New Jersey, after Dr. C. S. Stockton, of Newark, had read a very interesting paper entitled "Our Calling," the election of officers for the ensuing year took place. The result follows:

Dr. O. E. Peck, of Bridgeton, President; Dr. Charles P. Tuttle, of Camden, Vice-President; Dr. T. V. Smith, Jr., of Camden, Corresponding Secretary; Dr. A. K. Wood, Camden, Recording Secretary; Dr. Mary A. Morrison, Treasurer.

*Executive Committee.*—Dr. A. Irwin, Chairman; Drs. J. E. Duffield, Camden; J. G. Halsey, E. E. Bower, W. A. Jacquette, and A. B. Dewees, Camden.

A. IRWIN,
*Chairman Executive Committee.*

---

### JEFFERSON COUNTY DENTAL SOCIETY.

THE sixth annual meeting of the Jefferson County Dental Society was held in Watertown, N. Y., December 10, 1900. It was largely attended by dentists of Central and Northern New York. Drs. F. M. Willis, of Ithaca, Charles H. Barnes, of Syracuse, and I. C. Curtis, of Fulton, N. Y., gave very interesting clinics. These, together with President George B. Parker's address, and papers by Drs. C. W. Howard and G. R. Danforth, of Watertown, made the most successful and instructive meeting in the Society's history.

The election of officers for the ensuing year resulted as follows: President, D. A. Scobie, Ogdensburg; Vice-President. G. R. Danforth, Watertown; Secretary and Treasurer, R. F. Caster, Watertown.

E. E. HARRINGTON,
*Secretary.*

WATERTOWN, N. Y.

---

### COLORADO STATE DENTAL ASSOCIATION.

THE fifteenth annual meeting of the Colorado State Dental Association will be held in Denver, Tuesday, Wednesday, and Thursday, July 9, 10, and 11, 1901.

H. F. HOFFMAN,
*Secretary.*

DENVER, COL.

